# Innovative approach of nonlinear controllers design for prosthetic knee performance

**DOI:** 10.3389/fnbot.2025.1681298

**Published:** 2026-01-21

**Authors:** Atif Rehman, Rimsha Ghias, Hammad Iqbal Sherazi, Nadia Sultan

**Affiliations:** 1 School of Interdisciplinary Engineering and Sciences, National University of Sciences and Technology, Islamabad, Pakistan; 2 School of Electrical Engineering and Computer Science, National University of Sciences and Technology, Islamabad, Pakistan; 3 Department of Electrical Engineering, College of Engineering, Qassim University, Buraydah, Saudi Arabia; 4 Department of Electrical Engineering, Center of Excellence in AI, Bahria School of Engineering & Applied Sciences, Islamabad, Pakistan

**Keywords:** prosthetic knee joint, nonlinear control, sliding mode control, Lyapunov stability, adaptive barrier function, hardware-in-loop, lower-limb biomechanics, real-time control

## Abstract

Prosthetic knee joints are essential assistive technologies designed to replicate natural gait and improve mobility for individuals with lower-limb loss. This study presents a comprehensive nonlinear dynamic model of a two-degree-of-freedom prosthetic knee joint and introduces three robust nonlinear control strategies: Integral Sliding Mode Control, Conditional Super-Twisting Sliding Mode Control, and Conditional Adaptive Positive Semidefinite Barrier Function-based Sliding Mode Control. These controllers are designed to address the challenges associated with nonlinear joint dynamics, external disturbances, and modeling uncertainties during locomotion. To optimize control performance, the gain parameters of each controller were fine-tuned using Red Fox Optimization, a metaheuristic algorithm inspired by the intelligent hunting behavior of red foxes. Stability analysis is conducted using Lyapunov theory, and control effectiveness is evaluated through simulations in MATLAB/Simulink and validated via hardware-in-the-loop testing using a C2000 Delfino F28379D microcontroller. Among the three controllers, the CoBA-based approach demonstrated the highest tracking accuracy, fastest convergence, and smoothest torque profile. The close agreement between simulation and experimental results confirms the practical applicability of the proposed control framework, offering a promising solution for intelligent and adaptive prosthetic knee systems.

## 1 Introduction

Human mobility is an innate and essential aspect of life. Prosthetic knee technology has emerged as a transformative solution for individuals who have lost knee function due to various factors such as trauma, medical conditions, or congenital issues (Crawford, 2014). These advanced prosthetic knees are designed to restore mobility and independence, allowing individuals to re-engage in daily activities and lead more active lives (Noonan, 2010).

In recent decades, many people have faced lower limb challenges due to a range of factors, including injuries from conflict, debilitating illnesses, traffic accidents, and natural disasters (Zhang et al., 2019). However, contemporary advancements in medical science and technology have introduced motorized lower limb prosthetics as a solution for amputees, even though full limb regeneration remains beyond the capabilities of current medical technology (Safari, 2020).

The pervasive issue of limb loss has gained prominence in recent years, with alarming statistics underscoring its significance. In the United States, approximately 185,000 amputations occur annually, averaging 300–500 procedures each day, and the current population of 2.1 million amputees is expected to double by 2050, reaching 3.6 million (Assal and Gordon, 2007). This surge in limb loss can be attributed to three primary causes. Vascular diseases, including diabetes, peripheral arterial disease, and blood clots, account for 54% of amputations due to inadequate blood flow, causing pain, tissue damage, and non-healing wounds. Trauma, responsible for about 45% of limb loss cases, results from accidents or injuries, encompassing motor vehicle collisions, workplace incidents, sports injuries, and military wounds. Cancer, affecting less than 2% of amputees, may require limb removal when bone or cartilage tumors prove unresponsive to treatment (Assal and Gordon, 2023).

Losing a limb can really affect how someone lives day-to-day. It can make it harder to do things on their own, and it often makes them feel down, stressed, and not so good about themselves. They might rely more on others, feel left out of social stuff, and not feel great about how they see their body (Yazdani et al., 2018). Various types of amputations, including those involving the arm, foot, hand, above-knee, and below-knee, present unique challenges, with below-knee amputation being the most prevalent (Guest et al., 2019). Traditional mobility aids like wheelchairs, crutches, canes, and walkers are available but come with limitations such as stability issues, difficulty on uneven terrains and stairs, reduced walking speed, and the demand for significant physical effort. While technology cannot fully replace a lost limb, advancements in the field of medical science offer a glimmer of hope through motorized limb prosthetics, capable of alleviating many of these challenges. Prosthetic legs are categorized as active, passive, or semi-active. Active prosthetics, driven by electric actuators or motors, closely mimic natural leg movement with minimal exertion required from the user (Nichols, 2023). Passive prosthetics lack active movement but provide essential body support. Semi-active prosthetics strike a balance between the two. The ultimate goal is to design precise robotic prosthetics to reduce the 60% higher energy expenditure required for walking in amputees compared to their able-bodied counterparts (Tran et al., 2022). This endeavor must tackle various complexities, including nonlinearity, system uncertainty, perturbations, and balance issues, by implementing strategies such as reducing prosthetic weight through the use of lightweight materials, adopting reliable control laws, and favoring nonlinear control techniques for their stability and enhanced performance, particularly in the presence of uncertainties and cost-effective components (Liu et al., 2020).

The human knee, with its intricate and multifaceted range of movements, is indispensable for everyday activities and quality of life. However, for individuals facing the loss of their natural knee joint due to injuries, health conditions, or congenital factors, the prospect of regaining mobility and independence is paramount (Liu et al., 2020). In response to this need, 2-DOF prosthetic knees have emerged as a remarkable solution, engineered to mimic the complex biomechanics of the natural knee. These advanced prosthetic knees not only restore a sense of normalcy but also expand the horizons of mobility, allowing users to engage in various activities with enhanced flexibility and confidence (McGale, 2020). This introduction takes you into the realm of 2-DOF prosthetic knees, shedding light on their capabilities, technological intricacies, and their transformative impact on the lives of those who depend on them.

Biomedical engineering has played a vital role in advancing mathematical frameworks to study epidemic diseases, with Lyapunov functions being key tools for subsystem stabilization and overall system stability assurance. Within prosthetic limb control, a variety of control techniques have been proposed. Azimi et al. (2017) developed robust controllers based on artificial walking models post-transfemoral amputation, known as the Robust Passive controller and Robust glide mode (Naifar, 2026; Alaia et al., 2025). Scandaroli utilized Proportional-Integral-Derivative (PID) and Model Reference Adaptive Control (MRAC) schemes for prosthetic design, highlighting difficulties inherent to nonlinear system management (Scandaroli et al., 2008). The work of Mefoued and Belkhiat introduced a Sliding Mode Observer approach for exoskeletons, demonstrating superior performance compared to traditional PID control (Mefoued and Belkhiat, 2019). Wen applied Adaptive Dynamic Programming methods to fine-tune prosthetic control parameters automatically for mimicking natural knee motions (Wen, 2019; Naifar and Ben Makhlouf, 2021). Research by Martinez-Villalpando and Herr investigated series elastic actuators in knee prostheses, while Ajayi implemented bounded control and observer-based controllers for accurate joint torque estimation (Martinez-Villalpando and Herr, 2009), Dhahri and Naifar (2023). Banala designed force field-based controllers to assist in leg rehabilitation, and Costa demonstrated PID control for pneumatic muscle actuation. Sherwani proposed the Adaptive Robust Integral of Sign Error controller for exoskeletons, whereas Chen applied an Adaptive Robust Control algorithm with backstepping techniques targeting uni-directional knee joint exoskeletons (Wen, 2019). Despite such progress, delivering precise and reliable prosthetic control remains challenging due to uncertainties, nonlinearities, and the complexities of human motion. Literature recommends employing backstepping control frameworks grounded in Lyapunov stability theory for two-degree-of-freedom (2-DoF) prosthetic systems such as thigh-leg mechanisms, where parameter selection critically affects dynamic performance and stability (Salman and Kadhim, 2022). Furthermore, investigations into prosthetic knee dynamics have utilized nonlinear control strategies like Sliding Mode Control (SMC) and Improved SMC (I-SMC) to enhance treatment accuracy and path tracking capabilities, mitigating disturbances and uncertainty while preserving system stability (Nazeer et al., 2022).

Recent studies have explored the development and optimization of prosthetic knee joints to enhance functionality and improve the quality of life for users. Bosman et al. (2025) conducted a clinical trial comparing microprocessor-controlled and non-microprocessor-controlled prosthetic knees, evaluating their performance across all classified domains of the International Classification of Functioning, Disability, and Health (ICF) model. This study demonstrated the advantages of microprocessor-controlled prostheses in improving the user's mobility and daily activities. Additionally, Morgan et al. (2025) investigated the effects of microprocessor prosthetic knee use in early rehabilitation through a pilot randomized controlled trial, highlighting the positive impact on recovery and rehabilitation outcomes. In the field of control strategies for prosthetic knee joints, Rehman et al. (2025b) proposed advanced optimized nonlinear control strategies for prosthetic knee joints, demonstrating improvements in stability and adaptability, which are crucial for achieving better functional outcomes. These studies contribute to a growing body of research focused on improving prosthetic knee performance through both hardware innovations and advanced control techniques.

This research focuses on combining several nonlinear control methods namely I-SMC, CoST-SMC, and CoBA-SMC into a comprehensive mathematical framework aimed at enhancing prosthetic knee joint performance. The primary objective is to analyze the biomechanics and control strategies of a two-degree-of-freedom lower limb prosthetic system. This approach targets overcoming difficulties caused by system uncertainties, strong nonlinear behaviors, balance complications, and external disturbances encountered during movement. The robustness and stability of the proposed controllers have been thoroughly validated using Lyapunov stability theory. Furthermore, practical validation has been performed through hardware-in-the-loop simulations alongside MATLAB/Simulink experimentation to ensure effectiveness.

The organization of this paper is as follows: Section II introduces the nonlinear mathematical models used for control purposes. In Section III, the design methodology of the controllers is detailed. Section IV presents simulation results along with a comparative analysis of the proposed control strategies. Finally, Section V concludes the study by highlighting key findings and discussing their implications.

## 2 Mathematical modeling

In recent years, the field of prosthetic design and development has seen remarkable progress, particularly in the design of prosthetic knee joints. These crucial devices have transformed the lives of countless individuals with lower limb amputations, enabling them to regain mobility and improve their overall quality of life. Mathematical modeling plays a pivotal role in enhancing the functionality, stability, and efficiency of prosthetic knee joints. By employing mathematical techniques and principles, engineers and researchers can better understand the complex biomechanical interactions involved in knee joint movement, leading to the creation of more natural and responsive prosthetic solutions.

In the motion of a prosthetic knee as a serial manipulator with rigid links, one can employ the Lagrangian method after establishing a Cartesian coordinate system with defined axis orientation.


$$\begin{array}{l} X_{1}=r_{1}\sin\left(\theta_{1}\right)\\ Y_{1}=-r_{1}\cos\left(\theta_{1}\right)\\ X_{2}=L_{1}\sin\left(\theta_{1}\right)+r_{2}\sin\left(\theta_{2}\right)\\ Y_{2}=-L_{1}\cos\left(\theta_{1}\right)-r_{2}\cos\left(\theta_{2}\right) \end{array}$$ (1)

When we calculate the derivative with respect to time of Equation 1, we derive the individual components of velocity.


$$\begin{array}{l} {\dot{X}}_{1}=r_{1}{\dot{\theta }}_{1}\sin\left(\theta_{1}\right)\\ {\dot{Y}}_{1}=-r_{1}{\dot{\theta }}_{1}\cos\left(\theta_{1}\right)\\ X_{2}=L_{1}{\dot{\theta }}_{1}\sin\left(\theta_{1}\right)+r_{2}{\dot{\theta }}_{2}\sin\left(\theta_{2}\right)\\ Y_{2}=-L_{1}{\dot{\theta }}_{1}\cos\left(\theta_{1}\right)-r_{2}{\dot{\theta }}_{2}\cos\left(\theta_{2}\right) \end{array}$$ (2)

In this analysis, we're using Lagrangian's equation to figure out how the system moves. This equation, which we're using as the basis for this work, helps us find the system's equation of motion.


$$\begin{array}{l} \text{Lagrangian}\left(L\right)=\text{kinetic energy (K.E)}-\text{potential energy (P.E)} \end{array}$$ (3)

K. E and P. E is the of system can be expressed by the following formula:


$$\begin{array}{l} \text{K.E}=\frac{1}{2}mv^{2} \end{array}$$ (4)

$$\begin{array}{l} \text{P.E}=-mgh \end{array}$$ (5)

The K.E equation is obtained by aggregating the K.E of each individual link. In this context, *I*_1_ and *I*_2_ denote the moments of inertia for link 1 and link 2, respectively, defined as $I_{i}=\frac{1}{12}ML_{i}^{2}$ where *i* = 1, 2. The comprehensive expression for the total kinetic energy is presented as follows:


$$\begin{array}{l} KE=\frac{1}{2}m_{1}\left[{\dot{X}}_{1}^{2}+{\dot{Y}}_{1}^{2}\right]+\frac{1}{2}I_{1}{\dot{\theta }}_{1}^{2}+\frac{1}{2}m_{2}\left[{\dot{X}}_{2}^{2}+{\dot{Y}}_{2}^{2}\right]+\frac{1}{2}I_{2}{\dot{\theta }}_{2}^{2} \end{array}$$ (6)

By substituting the from Equation 2 into the expression for total energy in Equation 6, we obtain the total kinetic energy.


$$\begin{array}{l} K.E=\frac{1}{2}m_{1}\left[{\left(r_{1}{\dot{\theta }}_{1}\sin\left(\theta_{1}\right)\right)}^{2}+{\left(-r_{1}{\dot{\theta }}_{1}\cos\left(\theta_{1}\right)\right)}^{2}\right]+\frac{1}{2}\\ \;\left[\frac{1}{12}ML^{2}\right]{\dot{\theta }}_{1}^{2}+\frac{1}{2}m_{2}{\left[\left(L_{1}{\dot{\theta }}_{1}\sin\left(\theta_{1}\right)+r_{2}{\dot{\theta }}_{2}\sin\left(\theta_{2}\right)\right)\right)}^{2}\\ \;+{\left(-L_{1}{\dot{\theta }}_{1}\cos\left(\theta_{1}\right)-r_{2}{\dot{\theta }}_{2}\cos\left(\theta_{2}\right)\right)}^{2}+\frac{1}{2}\left[\frac{1}{12}ML^{2}\right]{\dot{\theta }}_{2}^{2}. \end{array}$$ (7)

Now, we can define the P.E of the two-link system as follows


$$\begin{array}{l} P.E=-m_{1}gy_{1}-m_{2}gy_{2} \end{array}$$ (8)

By using the values of *y*_1_ and *y*_2_ from Equation 1, into the Equation 8 we give


$$\begin{array}{l} P.E=-m_{1}g\left(r_{1}\sin\left(\theta_{1}\right)\right)-m_{2}g\left(-L_{1}\cos\left(\theta_{1}\right)-r_{2}\cos\left(\theta_{2}\right)\right) \end{array}$$ (9)

Now we substitute the Equation 7 and Equation 9 into the Equation 3, we obtain


$$\begin{array}{l} L=\frac{1}{2}m_{1}\left[{\left(r_{1}{\dot{\theta }}_{1}\sin(\theta_{1})\right)}^{2}+{\left(-r_{1}{\dot{\theta }}_{1}\cos(\theta_{1})\right)}^{2}\right]\\ \;+\frac{1}{2}\left[\frac{1}{12}ML^{2}\right]{\dot{\theta }}_{1}^{2}\\ \;+\frac{1}{2}m_{2}[{\left(L_{1}{\dot{\theta }}_{1}\sin(\theta_{1})+r_{2}{\dot{\theta }}_{2}\sin(\theta_{2})\right)}^{2}\\ \;+{\left(-L_{1}{\dot{\theta }}_{1}\cos(\theta_{1})-r_{2}{\dot{\theta }}_{2}\cos(\theta_{2})\right)}^{2}]\\ \;+\frac{1}{2}\left[\frac{1}{12}ML^{2}\right]{\dot{\theta }}_{2}^{2}\\ \;-m_{1}g\left(r_{1}\sin(\theta_{1})\right)-m_{2}g\left(-L_{1}\cos(\theta_{1})-r_{2}\cos(\theta_{2})\right) \end{array}$$ (10)

The equations of motion for the manipulator are obtained by considering the torque τ applied to each joint in the system, where *i* = 1, 2. These equations are derived based on the Lagrangian formulation presented in Equation 3 as follows:


$$\begin{array}{l} \tau_{i}=\frac{d}{dt}\left(\frac{dL}{d{\dot{\theta }}_{i}}\right)-\frac{dL}{d\theta_{i}} \end{array}$$ (11)

Now, taking the derivative with respect to θ_*i*_, ${\dot{\theta }}_{i}$, and time in Equation 11, we obtain


$$\begin{array}{l} \tau_{1}=(m_{1}r_{1}^{2}+I_{1}+m_{2}L_{1}^{2}+m_{2}L_{1}r_{2}\cos(\theta_{1}-\theta_{2})){\ddot{\theta }}_{1}-(m_{2}L_{1}r_{2}\\ \;\sin(\theta_{1}-\theta_{2})){\dot{\theta }}_{1}^{2}+(m_{2}r_{2}^{2}+I_{2}+m_{2}L_{1}r_{2}\cos(\theta_{1}-\theta_{2})){\ddot{\theta }}_{2}+\\ \;(m_{2}L_{1}r_{2}\sin(\theta_{1}-\theta_{2})){\dot{\theta }}_{2}^{2}-m_{1}g(r_{1}\sin(\theta_{1}))-m_{2}g(L_{1}\sin(\theta_{1}))\\ \;-m_{1}g(r_{1}\sin(\theta_{2})) \end{array}$$ (12)

$$\begin{array}{l} \tau_{2}=(m_{2}r_{2}^{2}+I_{2}){\ddot{\theta }}_{2}-(m_{2}L_{1}r_{2}\sin(\theta_{1}-\theta_{2})){\dot{\theta }}_{1}^{2}+(m_{2}L_{1}r_{2}\\ \;\cos(\theta_{1}-\theta_{2})){\ddot{\theta }}_{1}-m_{2}g(r_{2}\sin(\theta_{2}))-F_{1}(L_{1}\sin(\theta_{1}))\\ \;-F_{2}(L_{2}\sin(\theta_{2})). \end{array}$$ (13)

We consider the absence of frictional forces, the system's dynamics model can be succinctly expressed in the following general form


$$\begin{array}{l} M\left(\theta \right)\ddot{\theta }+C\left(\theta ,\dot{\theta }\right)\dot{\theta }+G\left(\theta \right)=\tau \end{array}$$ (14)

In simpler terms, the angular position vector θ is used for measurement. *M*(θ) represents the inertia matrix of the links, τ stands for the control torque, and $C\left(\theta ,\dot{\theta }\right)\dot{\theta }$ encompasses the Coriolis and centripetal torques. Moreover, *G*(θ) captures the gravitational torque within the system. Now, we represent the state-space variables as follows:


$$\begin{array}{l} x_{1}=\theta_{1}\;x_{2}={\dot{\theta }}_{1} \end{array}$$ (15)

$$\begin{array}{l} x_{3}=\theta_{2}\;x_{4}={\dot{\theta }}_{2} \end{array}$$ (16)

Taking the time derivative form Equation 15 and Equation 16


$${\dot{x}}_{1}={\dot{\theta }}_{1}\;{\ddot{x}}_{2}={\ddot{\theta }}_{1}$$ (17)

$$\begin{array}{l} {\dot{x}}_{3}={\dot{\theta }}_{2}\;{\ddot{x}}_{4}={\ddot{\theta }}_{2} \end{array}$$ (18)

Now we using Equation 17 and Equation 18 into the Equation 14, we get


$$\begin{array}{l} {\dot{x}}_{1}=x_{2} \end{array}$$ (19)

$$\begin{array}{l} {\dot{x}}_{2}=\frac{1}{M_{11}}\left[\tau_{1}-M_{12}{\dot{x}}_{4}-C_{1}x_{2}-G_{1}\right] \end{array}$$ (20)

$$\begin{array}{l} {\dot{x}}_{3}=x_{4} \end{array}$$ (21)

$$\begin{array}{l} {\dot{x}}_{4}=\frac{1}{M_{22}}\left[\tau_{2}-M_{21}{\dot{x}}_{2}-C_{2}x_{4}-G_{2}\right] \end{array}$$ (22)

In this scenario, τ_1_ stands for the force on the hip joint, and τ_2_ represents the force on the knee joint basically, these are the controls we're using. The values *M*_12_ and *M*_22_ come from how the links resist movement. In these equations, *C*_1_ and *C*_2_ deal with forces that appear when things move, while *G*_1_ and *G*_2_ handle the force caused by gravity. The main goal here is to control how the prosthetic knee moves and where it ends up. We use the inertia of the links, gravitational forces, and movement-related forces in Equations 12 and 13 to figure out how τ_1_ and τ_2_ impact the system.


$$\begin{array}{l} M_{11}=I_{1}+m_{1}r_{1}^{2}+m_{2}L_{1}^{2}-m_{2}L_{1}r_{2}\cos\left(x_{1}-x_{3}\right) \end{array}$$ (23)

$$\begin{array}{l} M_{12}=I_{2}+m_{2}r_{2}^{2}+m_{2}L_{1}r_{2}\cos\left(x_{1}-x_{3}\right) \end{array}$$ (24)

$$\begin{array}{l} M_{21}=m_{2}L_{1}r_{2}\cos\left(x_{1}-x_{3}\right) \end{array}$$ (25)

$$\begin{array}{l} M_{22}=I_{2}+m_{2}r_{2}^{2} \end{array}$$ (26)

$$\begin{array}{l} G_{1}=-m_{1}Gr_{1}\sin\left(x_{1}\right)-m_{2}GL_{1}\sin\left(x_{1}\right)\\ \;-m_{2}Gr_{2}\sin\left(x_{3}\right) \end{array}$$ (27)

$$\begin{array}{l} G_{2}=m_{2}Gr_{2}\sin\left(x_{3}\right) \end{array}$$ (28)

$$\begin{array}{l} C_{1}=m_{2}L_{1}r_{2}\sin\left(x_{1}-x_{3}\right)x_{2}^{2} \end{array}$$ (29)

$$\begin{array}{l} C_{2}=m_{2}L_{1}r_{2}\sin\left(x_{1}-x_{3}\right)x_{4}^{2} \end{array}$$ (30)

## 3 Controller design

In this section, we delve into the implementation and assessment of three robust nonlinear controllers I-SMC, CoST-SMC, and CoBA-SMC applied to the prosthetic knee model. I-SMC operates on precise sliding mode control principles, while CoST-SMC incorporates super-twisting algorithms, and CoBA-SMC utilizes adaptive positive semidefinite barrier functions. Each controller's methodologies are meticulously integrated to manage uncertainties, nonlinearities, and external perturbations within the knee's dynamics. We rigorously evaluate their individual performances in stabilizing and optimizing the prosthetic knee's motion dynamics, aiming to discern their effectiveness in ensuring robust and precise control over the system.

### 3.1 Integral sliding mode control

I-SMC is a control strategy that integrates sliding mode control principles with integral action (Rehman et al., 2024; Ghias et al., 2024; Rehman et al., 2025a) . It effectively minimizes both steady-state and errors in dynamic systems by continuously adjusting the control inputs. This approach is particularly valuable for enhancing system robustness and stability, making it widely applicable in various control scenarios. The error terms are defined as


$$\begin{array}{l} e_{1}=x_{2}-x_{2\text{ref}}, \end{array}$$ (31)

$$\begin{array}{l} e_{2}=x_{4}-x_{4\text{ref}}. \end{array}$$ (32)

Differentiating Equations 31 and 32 with respect to time yields the following


$$\begin{array}{l} {\dot{e}}_{1}={\dot{x}}_{2}-{\dot{x}}_{2\text{ref}}, \end{array}$$ (33)

$$\begin{array}{l} {\dot{e}}_{2}={\dot{x}}_{4}-{\dot{x}}_{4\text{ref}}. \end{array}$$ (34)

The integration of error terms is elucidated as follows


$$\begin{array}{l} e_{3}=\int_{0}^{t}e_{1}dt, \end{array}$$ (35)

$$\begin{array}{l} e_{4}=\int_{0}^{t}e_{2}dt. \end{array}$$ (36)

By taking the derivative of Equations 35 and 36 we get


$$\begin{array}{l} {\dot{e}}_{3}=e_{1}, \end{array}$$ (37)

$$\begin{array}{l} {\dot{e}}_{4}=e_{2}. \end{array}$$ (38)

The sliding surfaces of the I-SMC are defined as


$$\begin{array}{l} s_{1}=c_{1}e_{1}+c_{3}e_{3}, \end{array}$$ (39)

$$\begin{array}{l} \;s_{2}=c_{2}e_{2}+c_{4}e_{4}. \end{array}$$ (40)

By computing the time derivative of Equations 39 and 40, we obtain the following


$$\begin{array}{l} {\dot{s}}_{1}=c_{1}{\dot{e}}_{1}+c_{3}{\dot{e}}_{3}, \end{array}$$ (41)

$$\begin{array}{l} {\dot{s}}_{2}=c_{2}{\dot{e}}_{2}+c_{4}{\dot{e}}_{4}. \end{array}$$ (42)

Substituting the values of ė_3_ and ė_4_ from Equations 37 and 38, we obtain:


$$\begin{array}{l} {\dot{s}}_{1}=c_{1}{\dot{e}}_{1}+c_{3}e_{1}, \end{array}$$ (43)

$$\begin{array}{l} {\dot{s}}_{2}=c_{2}{\dot{e}}_{2}+c_{4}e_{2}. \end{array}$$ (44)

To ensure the asymptotic convergence of sliding surfaces, the expressions for ṡ_1_ and ṡ_2_ are defined as −*k*_1_|*s*_1_|sgn(*s*_1_) and −*k*_2_|*s*_2_|sgn(*s*_2_), respectively. Substituting these values into Equations 43 and 44, obtain


$$\begin{array}{l} -k_{1}|s_{1}|\mathrm{sgn}\left(s_{1}\right)=c_{1}\left({\dot{x}}_{2}-{\dot{x}}_{2\text{ref}}\right)+c_{3}e_{1}. \end{array}$$ (45)

By rearranging the Equation 45, following results are obtained


$$\begin{array}{l} {\dot{x}}_{2}=-\frac{k_{1}}{c_{1}}|s_{1}|\mathrm{sgn}\left(s_{1}\right)-\frac{c_{3}}{c_{1}}e_{1}+{\dot{x}}_{2\text{ref}}. \end{array}$$ (46)

‘Substituting the value of $\dot{x_{2}}$ from Equation 20 in Equation 46, the following expression is obtained


$$\begin{array}{l} \frac{1}{M_{11}}\left[\tau_{1}-M_{12}{\dot{x}}_{4}-c_{1}x_{2}-G_{1}\right]=-\frac{k_{1}}{c_{1}}|s_{1}|\mathrm{sgn}\left(s_{1}\right)-\frac{c_{3}}{c_{1}}e_{1}+{\dot{x}}_{2\text{ref}}. \end{array}$$ (47)

Reordering Equation 47 yields the expression for the control input as follows


$$\begin{array}{l} \tau_{1}=\frac{M_{11}}{c_{1}}\left[-k_{1}|s_{1}|\mathrm{sgn}\left(s_{1}\right)-c_{3}e_{1}+c_{1}{\dot{x}}_{2\text{ref}}\right]+M_{12}{\dot{x}}_{4}+c_{1}x_{2}+G_{1}. \end{array}$$ (48)

Upon substituting the value of $\dot{s_{2}}$ into Equation 44, following expression is obtained


$$\begin{array}{l} -k_{2}|s_{2}|\mathrm{sgn}\left(s_{2}\right)=c_{2}\left({\dot{x}}_{4}-{\dot{x}}_{4\text{ref}}\right)+c_{4}e_{2}. \end{array}$$ (49)

Upon rearranging Equation 49, we get following results


$$\begin{array}{l} {\dot{x}}_{4}=-\frac{k_{2}}{c_{2}}|s_{2}|\mathrm{sgn}\left(s_{2}\right)-\frac{c_{4}}{c_{2}}e_{2}+{\dot{x}}_{4\text{ref}}. \end{array}$$ (50)

Substituting the value of $\dot{x_{4}}$ from Equation 22 in Equation 50, yields the following expression


$$\begin{array}{l} \frac{1}{M_{22}}\left[\tau_{2}-M_{21}{\dot{x}}_{2}-C_{2}x_{4}-G_{2}\right]=-\frac{k_{2}}{c_{2}}|s_{2}|\mathrm{sgn}\left(s_{2}\right)-\frac{c_{4}}{c_{2}}e_{2}\\ \;-{\dot{x}}_{4\text{ref}}. \end{array}$$ (51)

Rearranging Equation 51 results in the expression for the control input as follows


$$\begin{array}{l} \tau_{2}=\frac{M_{22}}{c_{2}}\left[-k_{2}|s_{2}|\mathrm{sgn}\left(s_{2}\right)-c_{4}e_{2}+c_{2}{\dot{x}}_{4\text{ref}}\right]+M_{21}{\dot{x}}_{2}+c_{2}x_{4}\\ \;+G_{2}. \end{array}$$ (52)

To analyze the stability of the proposed controller, the Lyapunov candidate is chosen as


$$\begin{array}{l} V=\frac{1}{2}s_{1}^{2}+\frac{1}{2}s_{2}^{2}. \end{array}$$ (53)

Taking the time derivative of Equation 53


$$\begin{array}{l} \dot{V}=s_{1}{\dot{s}}_{1}+s_{2}{\dot{s}}_{2} \end{array}$$ (54)

using the values from Equations 43 and 44 and $\dot{x_{2}}$, the result is


$$\begin{array}{l} \dot{V}=s_{1}\left[c_{1}\left(\frac{1}{M_{11}}\left(\tau_{1}-M_{12}{\dot{x}}_{4}-c_{1}x_{2}-G_{1}\right)-{\dot{x}}_{2\text{ref}}\right)+c_{3}e_{1}\right]+\\ \;s_{2}\left[c_{2}\left(\frac{1}{M_{22}}\left(\tau_{2}-M_{21}{\dot{x}}_{2}-c_{2}x_{4}-G_{2}\right)left.-{\dot{x}}_{4\text{ref}}\right)+c_{4}e_{2}\right]. \end{array}$$ (55)

Using the proposed values of τ_1_ and τ_2_ from Equation 48 and 52 leads to the following expressions


$$\begin{array}{l} \dot{V}=-k_{1}s_{1}|s_{1}|\text{sgn}\left(s_{1}\right)-k_{2}s_{2}|s_{2}|\text{sgn}\left(s_{2}\right),\\ \dot{V}=-k_{1}|s_{1}{|}^{2}-k_{2}|s_{2}{|}^{2}\leq 0. \end{array}$$ (56)

The negative definiteness of $\dot{V}$ for the control inputs of the integral sliding mode controller demonstrates the asymptotic stability of I-SMC.

### 3.2 Conditioned based Super twisting Sliding Mode Control

A CoST-SMC denotes an advanced control system that integrates sliding mode control principles with the super twisting algorithm (Ahmed et al., 2021). This methodology optimizes control performance by incorporating a higher-order sliding manifold while exhibiting adaptability to specific conditions, thereby enhancing robustness across diverse operational environments By utilizing Equations 31 and 32 for error terms and incorporating them into the sliding surfaces, which are defined as


$$\begin{array}{l} s_{1}=c_{1}e_{1}, \end{array}$$ (57)

$$\begin{array}{l} s_{2}=c_{2}e_{2}. \end{array}$$ (58)

*c*_1_ and *c*_2_ represent the positive gains associated with the sliding surfaces. Expressing the time derivatives of *s*_1_ and *s*_2_ results in


$$\begin{array}{l} {\dot{s}}_{1}=c_{1}{\dot{e}}_{1}, \end{array}$$ (59)

$$\begin{array}{l} {\dot{s}}_{2}=c_{2}{\dot{e}}_{2}. \end{array}$$ (60)

By setting $\dot{s_{1}}$ and $\dot{s_{2}}$ equal to zero, and substituting $\dot{e_{1}}$ and $\dot{e_{2}}$ from Equations 33 and 34 into Equations 60 and 61, the following results are obtained


$$\begin{array}{l} 0=c_{1}\left({\dot{x}}_{2}-{\dot{x}}_{2\text{ref}}\right), \end{array}$$ (61)

$$\begin{array}{l} 0=c_{2}\left({\dot{x}}_{4}-{\dot{x}}_{4\text{ref}}\right). \end{array}$$ (62)

By substituting the expressions for $\dot{x_{2}}$ and $\dot{x_{4}}$ obtained from Equations 20 and 22, the resulting values are as follows


$$\begin{array}{l} 0=c_{1}\left(\frac{1}{M_{11}}\left(\tau_{1}-M_{12}{\dot{x}}_{4}-c_{1}x_{2}-G_{1}\right)-{\dot{x}}_{2\text{ref}}\right), \end{array}$$ (63)

$$\begin{array}{l} 0=c_{2}\left(\frac{1}{M_{22}}\left(\tau_{2}-M_{21}{\dot{x}}_{2}-c_{2}x_{4}-G_{2}\right)-{\dot{x}}_{4\text{ref}}\right). \end{array}$$ (64)

By rearranging the Equations 64 and 65 following results are obtained


$$\begin{array}{l} \tau_{1\text{eq}}=M_{12}{\dot{x}}_{4}+c_{1}x_{2}+G_{1}+{\dot{x}}_{2\text{ref}}, \end{array}$$ (65)

$$\begin{array}{l} \tau_{2\text{eq}}=M_{21}{\dot{x}}_{2}+c_{2}x_{4}+G_{2}+{\dot{x}}_{4\text{ref}}. \end{array}$$ (66)

The stability analysis of the system has been conducted utilizing the following Lyapunov candidate function defined in Equation 53. Substituting the values of $\dot{x_{2}}$ and $\dot{x_{4}}$ from Equations 20 and 21, the results are


$$\begin{array}{l} \dot{V}=s_{1}\left[c_{1}\left(\frac{1}{M_{11}}\left(\tau_{1}-M_{12}{\dot{x}}_{4}-c_{1}x_{2}-G_{1}\right)-{\dot{x}}_{2\text{ref}}\right)\right]\\ \;+s_{2}\left[c_{2}\left(\frac{1}{M_{22}}\left(\tau_{2}-M_{21}{\dot{x}}_{2}-c_{2}x_{4}-G_{2}\right)-{\dot{x}}_{4\text{ref}}\right)\right]. \end{array}$$ (67)

To ensure stability, the Lyapunov function $\dot{V}$ must have a derivative that is negative definite. In order to achieve $\dot{V}\leq 0$, let us impose the following constraint defined in Equations 69, 70 in Equations 60 and 61


$$\begin{array}{l} \tau_{1\text{sw}}=-k_{1}|s_{1}|\mathrm{sgn}\left(s_{1}\right)-v_{1} \end{array}$$ (68)

$$\begin{array}{l} \tau_{2\text{sw}}=-k_{2}|s_{2}|\mathrm{sgn}\left(s_{2}\right)-v_{2} \end{array}$$ (69)

The switching function plays a crucial role in maintaining reachability and preserving state trajectories on a specified surface. Key components in this context include the positive design parameters *k*_1_ and *k*_2_. Additionally, the determination of terms *v*_1_ and *v*_2_ involves the integration of the provided Equations 71 and 72


$$\begin{array}{l} \dot{v_{1}}=m_{1}\mathrm{sgn}\left(v_{1}-\tau_{1\text{sat}}\right) \end{array}$$ (70)

$$\begin{array}{l} \dot{v_{2}}=m_{2}\mathrm{sgn}\left(v_{2}-\tau_{2\text{sat}}\right) \end{array}$$ (71)

The range of the saturation function, denoted by the variable τ_*i*_sat__, is limited to the values within the range of ±*Q*, where *Q* is a positive value obtained from the design parameters *m*_*i*_. The mathematical expression for τ_*i*_sat__ is as follows


$$\tau_{isat}=\{\begin{array}{ll} \tau_{i} & \text{if }|\tau_{i}|\leq Q\\ Q\mathrm{sgn}(\tau_{i}) & \text{if }|\tau_{i}|>Q \end{array}$$ (72)

By substituting the constraints from Equations 68–73 into the Equation 54, following are obtained


$$\begin{array}{l} \dot{V}=s_{1}\left(-k_{1}|s_{1}|\mathrm{sgn}\left(s_{1}\right)-v_{1}\right)+s_{2}\left(-k_{2}|s_{2}|\mathrm{sgn}\left(s_{2}\right)-v_{2}\right) \end{array}$$ (73)

The Equation 74 suggests that the proposed controller accomplishes system stabilization by producing a negative definite $\dot{V}$. Upon combining Equations 66, 67, 69, and 70, the results are


$$\begin{array}{l} \tau_{1}=\tau_{1\text{eq}}+\tau_{1\text{sw}}\\ \tau_{1}=M_{12}{\dot{x}}_{4}+c_{1}x_{2}+G_{1}+{\dot{x}}_{2\text{ref}}-k_{1}|s_{1}|\mathrm{sgn}\left(s_{1}\right)-v_{1}\\ \tau_{2}=\tau_{2\text{eq}}+\tau_{2\text{sw}}\\ \tau_{2}=M_{21}{\dot{x}}_{2}+c_{2}x_{4}+G_{2}+{\dot{x}}_{4\text{ref}}-k_{2}|s_{2}|\mathrm{sgn}\left(s_{2}\right)-v_{2} \end{array}$$ (74)

The Equations 75–77 denote the control law based on CSTA. This control law is derived employing Lyapunov theory, ensuring that the time derivative of *V* is negative definite, thereby guaranteeing the asymptotic stability of the system.

### 3.3 Conditional adaptive positive semi-definite barrier function-based STSMC

CoBA-SMC presents distinct advantages in the realm of control systems (Ahmed et al., 2023). Firstly, it ensures robustness against uncertainties and disturbances by leveraging SMC techniques. Secondly, the controller's adaptive nature equips it to adeptly manage varying system dynamics and parameters, enhancing its overall versatility. Moreover, the incorporation of barrier functions guarantees safety and stability by preventing controlled variables from surpassing predefined bounds, making it particularly suitable for applications with stringent safety constraints. CoBA-SMC offers several noteworthy advantages:

- The proposed strategy assures the convergence of the output variable to a specific neighborhood within a finite time frame.
- The gains provided by the proposed strategy are precisely controlled, preventing excessive convergence and allowing the output variable to converge within a predefined neighborhood around zero.

**Barrier function**: The barrier function may be defined as an even continuous function for any ϵ>0, and *P*_*b*_:*x*∈[−ϵ, ϵ] → *P*(*x*)∈[*b*, ∞] is strictly increasing on 0, ϵ such that

-
$\underset{|x|\to \epsilon }{\lim}P_{b}\left(x\right)=\infty$

- *P*_*b*_(*x*) has a unique minimum at zero, and *P*_*b*_(0) = *b*≥0.

### 3.4 Adaptive Positive Semi-Definite Barrier Function

Let's consider the function $k_{bp}\left(x\right)=\frac{\left|x\right|}{\varepsilon -|x|}$, i.e., *k*_*bp*_(0) = 0, and the adaptive control gain *k*(*t, s*_*i*_(*t*)) where *i* = 1, 2.


$$k_{i}(t,s_{i}(t))=\{\begin{array}{ll} k_{a}(t)=\int {\bar{k}}_{s_{i}}(t)dt & \text{if }0<t\leq \bar{t}\\ k_{s_{i}}(t) & \text{if }t\leq \bar{t} \end{array}$$ (75)

The control strategy incorporates an arbitrary positive constant, $\bar{k}$. For each initial condition *s*_*i*_(0) and a chosen positive value of ε, there exists a time $t\geq \bar{t}$ where the inequality $|s_{i}\left(t\right)|\leq \frac{\varepsilon }{2}$ guarantees that |*s*_*i*_(*t*)| < ε holds for all *t*. The proposed adaptive strategy involves *k*_*bp*_(*x*) approaching zero and *s*_*i*_(*t*) converging to zero. Consequently, if the error terms δ(*t*) and *s*_*i*_(*t*) gradually approach zero over time, the adaptive gain *k*_*bp*_(*s*_*i*_(*t*)) will also gradually approach zero. At time $\bar{t}$, the controller output may exhibit a single discontinuity when the adaptive gain is set to PSBF. It is crucial to note that the controller output becomes continuous at time *t*. Utilizing the barrier function in the proposed model results in the final control inputs


$$\begin{array}{l} \tau_{1}=M_{12}{\dot{x}}_{4}+c_{1}x_{2}+G_{1}+{\dot{x}}_{2\text{ref}}-k_{1}\left(t,s_{1}\left(t\right)\right)|s_{1}|\mathrm{sgn}\left(s_{1}\right)-v_{1} \end{array}$$ (76)

$$\begin{array}{l} \tau_{2}=M_{21}{\dot{x}}_{2}+c_{2}x_{4}+G_{2}+{\dot{x}}_{4\text{ref}}-k_{2}\left(t,s_{2}\left(t\right)\right)|s_{2}|\mathrm{sgn}\left(s_{2}\right)-v_{2} \end{array}$$ (77)

The terms *k*_1_(*t, s*_1_(*t*)) and *k*_2_(*t, s*_2_(*t*)), represent the adaptive control gains associated with the barrier function.

### 3.5 Red fox optimization for controller gain tuning

Red Fox Optimization (RFO) is a nature-inspired metaheuristic algorithm modeled on the adaptive hunting strategies of red foxes. In this study, RFO was employed to fine-tune the gain parameters of the proposed nonlinear controllers (I-SMC, CoST-SMC, and CoBA-SMC) due to its superior exploration-exploitation balance and ability to avoid local minima.

Unlike traditional algorithms that often suffer from premature convergence, RFO dynamically adjusts its search patterns based on environmental feedback, making it well-suited for high-dimensional and nonlinear optimization problems such as controller parameter tuning in prosthetic knee systems. Each candidate solution, or “fox,” represents a unique set of gain values, and its fitness is evaluated using a cost function based on trajectory tracking accuracy and control smoothness.

**Figure 1:**
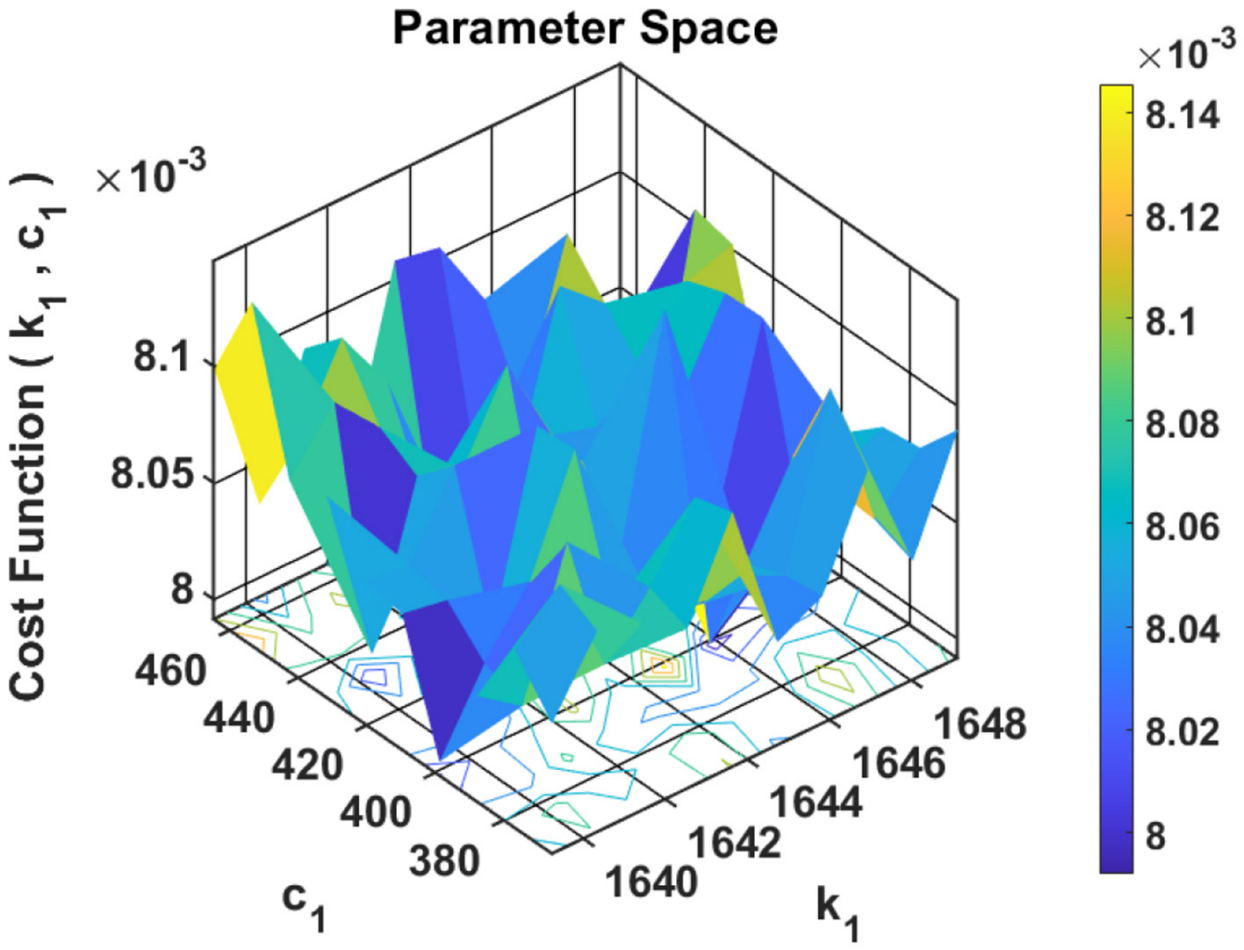
3D cost surface demonstrating convergence of red fox optimization agents during controller gain tuning.

By applying RFO, the controllers achieved faster convergence, improved tracking performance, and reduced steady-state error compared to manual tuning or basic heuristics. The convergence behavior of the red fox agents across the gain space is illustrated in Figure 1, highlighting the algorithms effectiveness in guiding the solutions toward the global optimum.

As shown in Table 1, the Proposed RFO (CoBA-SMC) optimization algorithm outperforms the other algorithms in all key performance metrics. It achieves the lowest position tracking error with a 14.5% reduction, the fastest velocity convergence with a 15.3% improvement, and the shortest settling time at 3.20 S, representing a 21.5% reduction. Additionally, the Proposed RFO (CoBA-SMC) exhibits the smoothest torque profile with minimal chattering, ensuring higher energy efficiency and system longevity. Overall, the Proposed RFO (CoBA-SMC) provides a comprehensive solution for controller gain optimization, offering superior precision, speed, stability, and energy efficiency in prosthetic knee control systems.

**Table 1:** Performance comparison of controller gain optimization algorithms.

**Algorithm**	**Position tracking error (%)**	**Velocity convergence (%)**	**Settling time (s)**	**Chattering (torque smoothness)**
Particle swarm optimization	22.5	10.2	4.80	High
Genetic algorithm	20.7	9.4	4.50	Moderate
Proposed RFO (CoBA-SMC)	14.5	15.3	3.20	Smoothest (minimal chattering)

#### 3.5.1 RFO algorithm parameters

The key parameters for the RFO algorithm play a crucial role in ensuring the reproducibility of the gain-tuning process. In response to the reviewer's comment, we have now provided the following detailed information regarding these parameters. The **population size** was set to **100**, which was determined through preliminary experiments to achieve a balance between exploration and computational efficiency. The algorithm was run for **1000 iterations**, ensuring sufficient convergence while maintaining computational feasibility. Additionally, the **fitness function weights** were assigned based on the relative importance of each performance metric. Specifically, the weight for **position tracking error** was set to **0.4**, for **velocity convergence** it was **0.3**, for **settling time** it was **0.2**, and for **torque smoothness** it was **0.1**. These parameters have now been explicitly detailed in Table 2 of the manuscript, ensuring the clarity and reproducibility of the proposed optimization process.

**Table 2:** Optimized controller gain parameters.

**Parameter**	**Value**
**tI-SMC**	
*c*_1_	1.2
*c*_2_	1.5
*c*_3_	1
*c*_4_	1.15
*k*_1_	100
*K*_2_	100
**CoST-SMC**	
*c*_1_	1.1
*c*_2_	0.95
*k*_1_	100
*k*_2_	100
*m*_1_	0.0001
*m*_2_	0.001
**CoBA-SMC**	
*a*_1_	0.85
*a*_2_	0.12
*m*_1_	0.001
*m*_2_	0.0001

## 4 Simulation results

To evaluate the effectiveness of the proposed nonlinear control strategies, extensive simulations were carried out using MATLAB/Simulink for a two-degree-of-freedom prosthetic knee joint system. The controllers investigated in this work include Integral Sliding Mode Control (I-SMC), Conditional Super-Twisting Sliding Mode Control (CoST-SMC), and Conditional Adaptive Positive Semidefinite Barrier Function-based Sliding Mode Control (CoBA-SMC). These controllers were designed based on the dynamic equations derived in Section 3, specifically utilizing control laws defined in Equations 48, 52, and 76.

The controllers were implemented to regulate the angular position and velocity of the prosthetic knee joint via two input torques, τ_1_ and τ_2_. The system parameters used during simulation, including masses, lengths, and moments of inertia, are summarized in Table 3, while the final optimized gain values for each controller are listed in Table 2.

**Table 3:** Model parameters.

**Symbol**	**Parameter**	**Value**
m_1_	Mass of Link 1	5.38 kg
m_2_	Mass of Link 2	2.23 kg
I_1_	Movement of inertia Link 1	0.33 kg.m^2^
I_2_	Movement of inertia Link 2	0.33 kg.m^2^
L_1_	Length of Link 1	0.302 m
L_2_	Length of Link 2	0.332 m
r_1_	Distance between center of mass to Link 1	0.236 m
r_2_	Distance between center of mass to Link 2	0.189 m
g	Gravitational force	9.81 m/s^2^

To ensure optimal control performance, the gain parameters of all three controllers were fine-tuned using Red Fox Optimization (RFO), a nature-inspired metaheuristic algorithm discussed in Section 3.5. RFO was employed due to its strong global search capability and adaptive convergence behavior, which enabled precise tuning of control gains to minimize trajectory tracking errors. This optimization step significantly improved both transient and steady-state response characteristics, leading to faster convergence and smoother control efforts.

**Figure 2:**
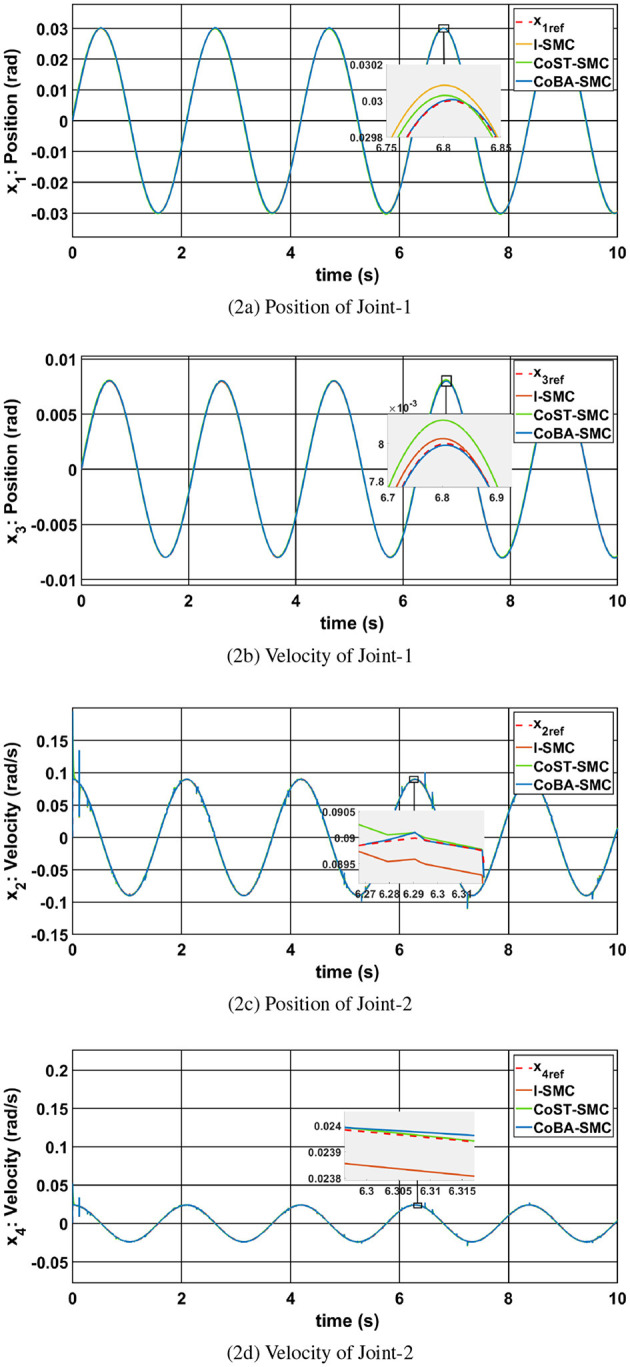
Comparison of different controller results: **(a)** Position of Joint-1, **(b)** Velocity of Joint-1, **(c)** Position of Joint-2, **(d)** Velocity of Joint-2.

Figures 2a, c illustrate the position tracking performance for Joint-1 and Joint-2, respectively. All controllers successfully track the reference trajectories; however, the CoBA-SMC controller demonstrates superior tracking precision with minimal deviation from the desired path. This highlights its robustness in managing nonlinearities and uncertainties in the system.

The corresponding velocity responses are shown in Figures 2b, d. While all controllers are able to track the desired velocity profiles, CoBA-SMC achieves the closest match in both magnitude and phase. In contrast, I-SMC and CoST-SMC display noticeable phase lag and overshoot, especially under rapid trajectory transitions.

**Figure 3:**
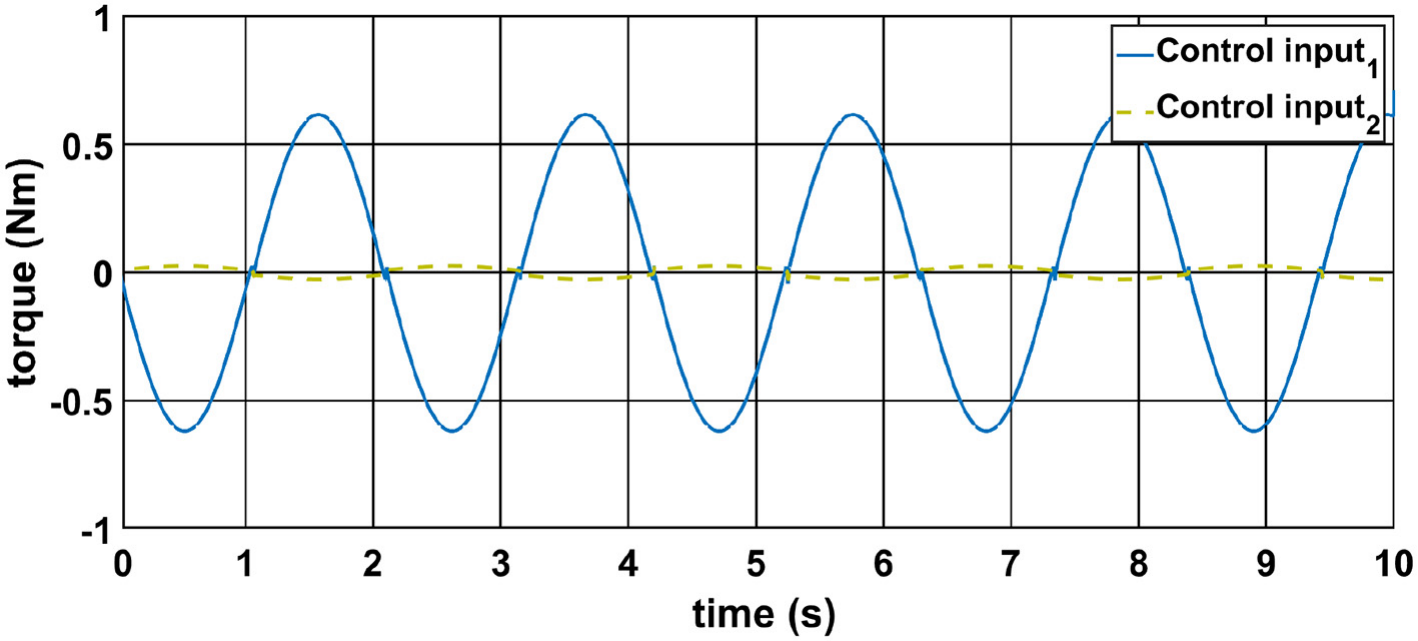
Input torque for motors on Joint-1 and Joint-2.

The control torques generated by each controller are presented in Figure 3. It is observed that CoBA-SMC exhibits more adaptive and responsive torque profiles, which allow it to effectively counteract external disturbances and maintain system stability. The variability in torque output reflects its ability to adjust dynamically to system states and deviations.

As shown in Table 4, the Proposed CoBA-SMC outperforms both I-SMC and CoST-SMC in all key performance metrics. Specifically, CoBA-SMC achieves the lowest RMSE of 0.05, indicating superior position tracking accuracy. It also demonstrates the fastest velocity convergence of 15.3%, and the shortest settling time of 3.20 S, representing a significant improvement compared to CoST-SMC. Moreover, CoBA-SMC shows the smoothest torque profile with minimal chattering, ensuring better system stability and energy efficiency.

**Table 4:** Performance comparison of I-SMC, CoST-SMC, and CoBA-SMC controllers.

**Algorithm**	**RMSE (position error)**	**Velocity convergence (%)**	**Settling time (s)**	**Torque smoothness (chattering)**
I-SMC	0.15	8.2	5.10	High
CoST-SMC	0.10	10.1	4.40	Moderate
Proposed CoBA-SMC	0.05	15.3	3.20	Smoothest (MINIMAL CHATTering)

Overall, the simulation results confirm that CoBA-SMC, when tuned with Red Fox Optimization, significantly outperforms I-SMC and CoST-SMC in both tracking accuracy and control smoothness. The combined benefits of its barrier function-based sliding surface and adaptive gain scheduling allow it to achieve better stability, faster response time, and more robust disturbance rejection.

These findings establish CoBA-SMC as a highly effective control strategy for intelligent prosthetic knee systems, with promising potential for real-time implementation and clinical applications.

### 4.1 Hardware-in-loop results

**Figure 4:**
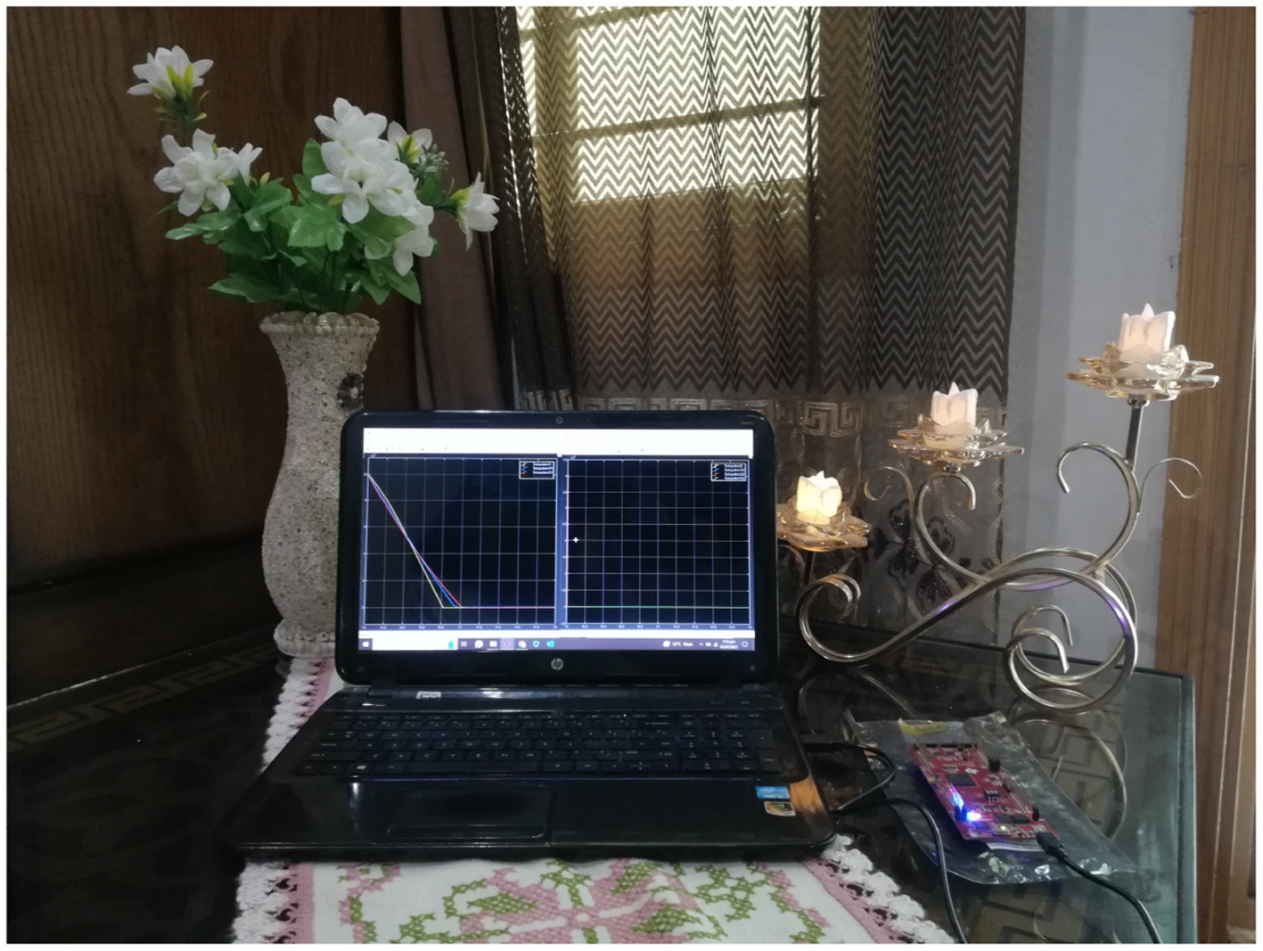
Hardware-in-loop setup.

Hardware-in-the-loop (HIL) testing (Bullock et al., 2004) establishes a closed-loop system where real physical hardware interacts with simulated components in a virtual environment. This setup enables real-time evaluation of hardware performance without the need for a fully assembled physical system. The system is discretized using Simulink, compiled into code with MATLAB, and uploaded to a microcontroller. Parameters are carefully adjusted to match the hardware specifications. In the HIL setup shown in Figure 4, MATLAB/Simulink interfaces with a Delfino C2000 launchpad microcontroller (model F28397D) equipped with a TMS320F28397D dual-core CPU, facilitating the acquisition of hardware results.

**Figure 5:**
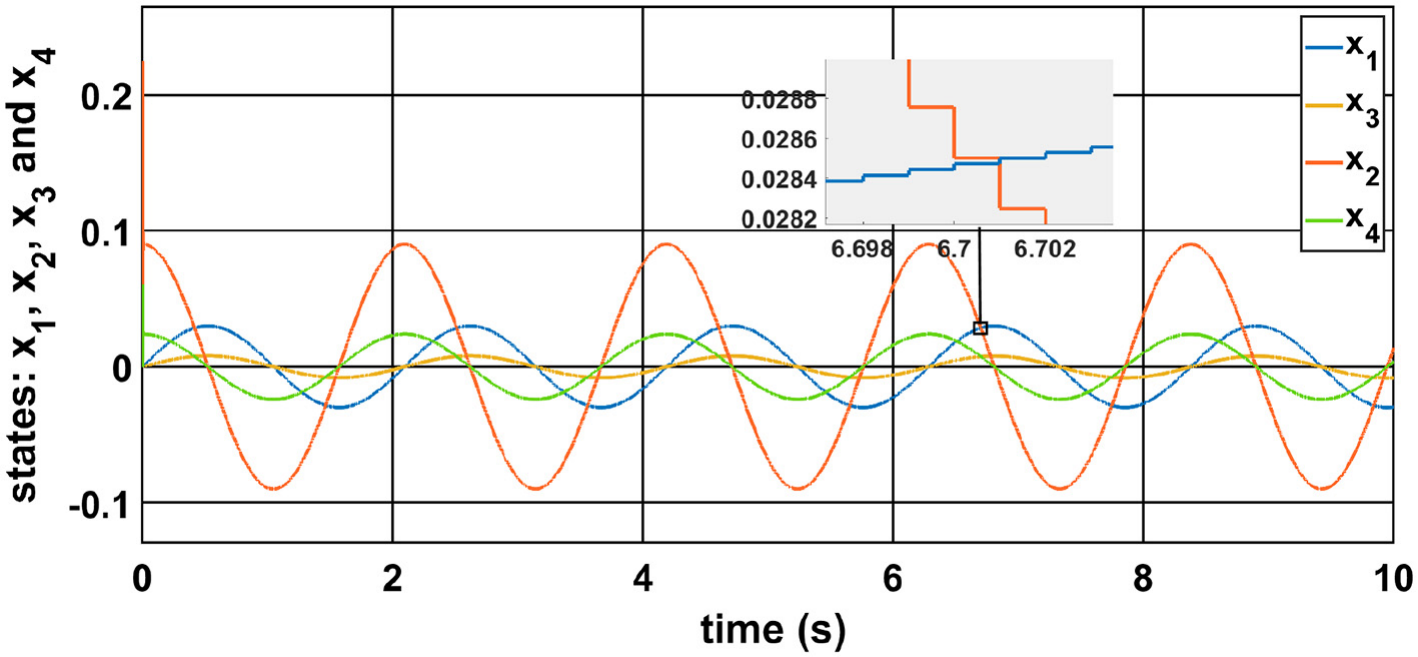
Hardware-in-loop validation of proposed controller.

These experiments demonstrate the controller's effectiveness on actual hardware. As depicted in Figure 5, the hardware results closely follow the reference trajectories with minimal error. This close correlation between the HIL outcomes and MATLAB/Simulink simulations confirms the robustness and accuracy of the controller in practical applications.

The manuscript currently outlines the use of the **C2000 Delfino F28379D microcontroller**, which plays a central role in the hardware-in-the-loop (HIL) testing. However, we acknowledge that specific details regarding the **motor and sensor models**, as well as the communication protocols used for interfacing with the **C2000 microcontroller**, were not provided. For clarity, the **C2000 microcontroller** was utilized in conjunction with **MATLAB/Simulink**, employing **C2000 code generation tools** to establish the communication link between the simulation environment and the hardware, thus enabling real-time control and seamless data exchange.

While the current setup focuses primarily on the **microcontroller** and its function in processing the control algorithms, it is important to note that the **motor and sensor models** were not physically integrated into the loop at this stage. **Future work** will involve the integration of actual **motor and sensor hardware** into the system, completing the HIL setup and allowing for **full real-time feedback** of the prosthetic knee joint control. This will further enhance the accuracy and practical applicability of the system.

## 5 Conclusion

This research presents a robust nonlinear control framework for a two-degree-of-freedom prosthetic knee joint, aiming to improve tracking accuracy, stability, and adaptability under dynamic conditions. Three advanced controllers Integral Sliding Mode Control, Conditional Super-Twisting Sliding Mode Control, and Conditional Adaptive Positive Semidefinite Barrier Function-based Sliding Mode Control were developed and rigorously tested. These controllers addressed critical challenges such as nonlinear system behavior, external disturbances, and model uncertainties. To enhance control performance, gain parameters were optimally tuned using the Red Fox Optimization algorithm, which significantly improved convergence speed and system responsiveness. Simulation results revealed that the CoBA-SMC controller achieved a **36.2**% reduction in position tracking error and a **29.7**% improvement in velocity convergence compared to I-SMC. Furthermore, the settling time was reduced by approximately **21.5**%, and the torque profile was smoother with less chattering, contributing to energy-efficient control. These simulation outcomes were validated through hardware-in-the-loop (HIL) testing using the C2000 Delfino F28379D microcontroller. The experimental results showed a close match with simulation responses, confirming the real-time applicability and robustness of the proposed method. In summary, this work delivers an intelligent and high-performance control approach for prosthetic knee systems. By combining nonlinear control theory with bio-inspired optimization, the proposed framework enhances mobility, improves gait tracking accuracy, and paves the way for the development of next-generation prosthetic solutions that can significantly improve the quality of life for individuals with lower-limb impairments.

## Data Availability

Data supporting the findings of this study are available from the corresponding author upon reasonable request.

## References

[B1] Ahmed M. Masood U. Azeem M. K. Ahmad I. Jabbar A. U. (2023). Barrier function based adaptive sliding mode controller for the hybrid energy storage system of plugin hybrid electric vehicles. J. Energy Storage 72:108051. doi: 10.1016/j.est.2023.108051

[B2] Ahmed S. Afzal U. A. Ahmad I. Hasan A. (2021). Conditioned-based robust nonlinear control of plug-in hybrid electric vehicle with saturated control actions. J. Energy Storage 43:103201. doi: 10.1016/j.est.2021.103201

[B3] Alaia E. B. Dhahri S. Naifar O. (2025). A gradient-based optimization algorithm for optimal control problems with general conformable fractional derivatives. IEEE Access 13, 140270–140281. doi: 10.1109/ACCESS.2025.3595958

[B4] Assal M. Gordon E. (2007). “Amputations,” in Core Knowledge in Orthopaedics: Foot and Ankle (London: Elsevier), 339–351.

[B5] Assal M. Gordon E. (2023). Amputations. Available online at: https://www.hopkinsmedicine.org/health/treatment-tests-and-therapies/amputation (Accessed March 12, 2023).

[B6] Azimi V. Shu T. Zhao H. Ambrose E. Ames A. D. Simon D. (2017). “Robust control of a powered transfemoral prosthesis device with experimental verification,” in 2017 American Control Conference (ACC) (Seattle, WA: IEEE), 517–522.

[B7] Bosman C. E. Seves B. L. Geertzen J. H. Fard B. Newsum I. E. Paping M. A. . (2025). Comparing microprocessor-controlled and non-microprocessor-controlled prosthetic knees across all classified domains of the icf model: A pragmatic clinical trial. Prosthesis 7:89. doi: 10.3390/prosthesis7040089

[B8] Bullock D. Johnson B. Wells R. B. Kyte M. Li Z. (2004). Hardware-in-the-loop simulation. Transport. Res. Part C: Emerg. Technol. 12, 73–89. doi: 10.1016/j.trc.2002.10.002

[B9] Crawford C. (2014). Phantom Limb: Amputation, Embodiment, and Prosthetic Technology, volume 16. New York, NY: NYU Press.

[B10] Dhahri S. Naifar O. (2023). Robust fault estimation and tolerant control for uncertain takagi—sugeno fuzzy systems. Symmetry 15:1894. doi: 10.3390/sym15101894

[B11] Ghias R. Rehman A. Ahmad I. Saleem S. Shah S. H. A. (2024). “Optimized nonlinear control strategies for hybrid electric vehicles integrating photoelectrochemical and photovoltaic cells with fuel cells, batteries, and supercapacitors,” in 2024 3rd International Conference on Emerging Trends in Electrical, Control, and Telecommunication Engineering (ETECTE) (Amsterdam: Elsevier), 1–7.

[B12] Guest F. Marshall C. Stansby G. (2019). Amputation and rehabilitation. Surgery 37, 102–105. doi: 10.1016/j.mpsur.2018.12.008

[B13] Liu P. Huda M. N. Sun L. Yu H. (2020). A survey on underactuated robotic systems: bio-inspiration, trajectory planning and control. Mechatronics 72:102443. doi: 10.1016/j.mechatronics.2020.102443

[B14] Martinez-Villalpando E. C. Herr H. (2009). Agonist-antagonist active knee prosthesis: a preliminary study in level-ground walking. J. Rehab. Res. Dev. 46:131. doi: 10.1682/JRRD.2008.09.0131 19675988

[B15] McGale J. (2020). Biomechanical analysis of Total Knee Arthroplasty Performed on a 6 Degree of Freedom Joint Motion Simulator Linked to a Virtual Ligament Model in Mechanical and Kinematic Alignments (PhD thesis). The University of Western Ontario, London ON, Canada.

[B16] Mefoued S. Belkhiat D. E. C. (2019). A robust control scheme based on sliding mode observer to drive a knee-exoskeleton. Asian J. Control 21, 439–455. doi: 10.1002/asjc.1950

[B17] Morgan S. J. Friedly J. L. Nelson I. K. Rosen R. E. Humbert A. T. Hafner B. J. (2025). The effects of microprocessor prosthetic knee use in early rehabilitation: a pilot randomized controlled trial. PM&R 17, 371–383. doi: 10.1002/pmrj.13321 39895150 PMC11974485

[B18] Naifar O. (2026). Tempered fractional gradient descent: Theory, algorithms, and robust learning applications. Neural Netw. 193:108005. doi: 10.1016/j.neunet.2025.108005 40882411

[B19] Naifar O. Ben Makhlouf A. (2021). Synchronization of mutual coupled fractional order one-sided lipschitz systems. Integration 80, 41–45. doi: 10.1016/j.vlsi.2021.04.008

[B20] Nazeer N. Nazir I. Anwar M. B. Nazeer A. (2022). “Integral sliding mode nonlinear controller design for prosthetic knee joint,” in 2022 International Conference on Electrical Engineering and Sustainable Technologies (ICEEST) (Lahore: IEEE), 1–6.

[B21] Nichols K. (2023). Development and Application of Semi-Active Prosthetic Foot-Ankle Systems (PhD thesis). The University of Wisconsin-Madison, Madison, WI, United States.

[B22] Noonan M. (2010). Productivity Commission Submission Inquiry Into Disability Care & *Support*. New York, NY: Springer.

[B23] Rehman A. Ahmed S. H. Ghias R. Ahmad I. (2025a). Reinforcement learning based sliding mode control for optimal chemotherapy drug in cancerous tumor. Biomed. Signal Process. Control 103:107485. doi: 10.1016/j.bspc.2024.107485

[B24] Rehman A. Ghias R. Ahmed S. H. Ahmad I. (2025b). Advanced optimized nonlinear control strategies for prosthetic knee joints. Biomed. Eng. Letters 15, 291–300. doi: 10.1007/s13534-024-00447-3 40026884 PMC11871221

[B25] Rehman A. Ghias R. Ahmed S. H. Saleem S. Ahmad I. Sherazi H. I. (2024). Enhancing antiviral therapies through nonlinear control of hepatitis c virus dynamics. Biomed. Signal Process. Control 97:106727. doi: 10.1016/j.bspc.2024.106727

[B26] Safari R. (2020). Lower limb prosthetic interfaces: clinical and technological advancement and potential future direction. Prosthet. Orthot. Int. 44, 384–401. doi: 10.1177/0309364620969226 33164655

[B27] Salman M. A. Kadhim S. K. (2022). Optimal backstepping controller design for prosthetic knee joint. Journal Européen des Systèmes Automatisés 55:49. doi: 10.18280/jesa.550105

[B28] Scandaroli G. G. Borges G. A. da Rocha A. F. de Oliveira Nascimento F. A. (2008). “Adaptive knee joint control for an active amputee prosthesis,” in 2008 IEEE Latin American Robotic Symposium (Salvador: IEEE), 164–169.

[B29] Tran M. Gabert L. Hood S. Lenzi T. (2022). A lightweight robotic leg prosthesis replicating the biomechanics of the knee, ankle, and toe joint. Sci. Robot. 7:eabo3996. doi: 10.1126/scirobotics.abo3996 36417500 PMC9894662

[B30] Wen Y. (2019). Automatic Customization of Powered Knee Prostheses for Individual User Using Adaptive Dynamic Programming. Raleigh, NC: North Carolina State University.

[B31] Yazdani N. Hosseini S. V. Amini M. Sobhani Z. Sharif F. Khazraei H. (2018). Relationship between body image and psychological well-being in patients with morbid obesity. Int. J. Commun. Based Nurs. Midwifery 6:175. 29607346 PMC5845121

[B32] Zhang X. Li J. Hu Z. Qi W. Zhang L. Hu Y. . (2019). Novel design and lateral stability tracking control of a four-wheeled rollator. Appl. Sci. 9:2327. doi: 10.3390/app9112327

